# Optimizing Gun Drilling Parameters for Oxygen-Free Copper Using Response Surface Methodology and Genetic Algorithm

**DOI:** 10.3390/ma18163913

**Published:** 2025-08-21

**Authors:** Xiaolan Han, Hailong Wang, Yazhou Feng, Shengdun Zhao

**Affiliations:** 1 Mechanical Engineering College, Xi’an Shiyou University, Xi’an 710065, China; pinbo08@163.com (H.W.); asian5921@126.com (Y.F.); 2 Shaanxi Shenkong Zhiyue Technology Co., Ltd., Xi’an 710032, China; 3 State Key Laboratory for Mechanical Behavior of Materials, Xi’an Jiaotong University, Xi’an 710049, China

**Keywords:** oxygen-free copper, gun drilling, response surface methodology-genetic algorithm

## Abstract

To improve chip removal efficiency and drilling performance in oxygen-free copper, a multi-objective optimization of gun drilling process parameters was conducted using a response surface methodology and a genetic algorithm. The Box–Behnken Design (BBD) response surface analysis method was employed to evaluate the effects of feed rate, cutting speed, and cutting fluid pressure on the chip evacuation coefficient and chip volume ratio. Experimental results indicate that among the three factors, the feed rate has the most significant influence, followed by the cutting speed and the cutting fluid pressure. Additionally, the interaction between the cutting speed and the cutting fluid pressure notably impacts both chip evacuation and chip volume ratio. Using response surface modeling, a three-dimensional predictive model was developed. Based on this fitted model, optimal gun drilling parameters were identified through genetic algorithm optimization, minimizing the chip evacuation coefficient and chip volume ratio to achieve an optimized machining configuration. The optimal drilling parameters were identified as a feed rate of 0.019 mm/r, a spindle speed of 47.1 m/min, and a cutting fluid pressure of 2.4 MPa. Under these conditions, a chip evacuation coefficient of 3.2951 and a chip volume ratio of 3.3345 were achieved. The resulting chips predominantly exhibited a C-shaped morphology, accompanied by smooth and efficient evacuation.

## 1. Introduction

The drilling process for oxygen-free copper plays a crucial role in various industrial applications due to the material’s exceptional electrical and thermal conductivity, corrosion resistance, and workability [1]. However, its high plasticity causes the formation of long chips during drilling, leading to difficult chip evacuation, severe tool wear, and compromised machining quality. Chip morphology significantly affects chip evacuation efficiency and deformation minimization, directly influencing machining stability, tool longevity, and surface integrity. Effective control of chip morphology is essential for optimizing chip removal.

The primary factors influencing chip morphology in drilling include tool geometry, cutting parameters, and cutting fluid conditions [2]. Among these, cutting parameters and cutting fluid characteristics are directly adjustable on-site. Naruki studied the effect of feed rate on chip evacuation in brass drilling, finding that higher feed rates produced long-pitch chips, whereas very low rates generated thin chips, improving evacuation and preventing flute clogging [3]. Altharan et al. investigated feed rate and spindle speed in deep-hole drilling of 50# steel (SAE/AISI 1050), identifying feed rate as the dominant factor in forming C-shaped chips and establishing a chip evacuation coefficient threshold of less than 10 for optimized parameters [4]. Habib et al. observed that increasing the feed rate and decreasing the spindle speed resulted in short, segmented, and discontinuous chips in dry drilling of Al7075 [5]. Lou examined deep-hole gun drilling of 304 stainless steel, revealing that higher spindle speed and feed rate increased chip curl radius, while elevated coolant pressure significantly enhanced deformation, particularly in spiral chip formation [6]. Additionally, greater coolant pressure promotes chip fragmentation by exerting stronger mechanical forces during formation [7], yielding smaller chip dimensions (diameter and length) [8]. This fragmentation, combined with a reduced curl radius under optimized coolant conditions [9], substantially improves chip evacuation efficiency while simultaneously reducing cutting temperatures and flashing effects.

Industry experience confirms that chip morphology control is vital for efficient evacuation. However, quantitative metrics correlating process parameters—such as feed rate, spindle speed, and cutting fluid properties—to chip morphology remain limited, necessitating reliance on empirical judgment and experimental trials [10]. To address this gap, Response Surface Methodology (RSM) has emerged as a viable optimization strategy. RSM enables the construction of empirical polynomial models by systematically designing experimental points, thereby establishing quantitative relationships between process parameters and response variables [11]. However, for highly nonlinear, discontinuous problems with multiple local optima or complex parameter interactions, lower-order polynomial models may inadequately represent the true response surface. Consequently, hybrid optimization techniques are often employed [12,13], including RSM-GA, RSM-FEA-GA, and ANN-GA. Among these, the RSM-GA approach has gained prominence as a robust and widely adopted method, particularly in the optimization of machining parameters in recent studies [14,15]. The Pareto optimal solutions generated by Genetic Algorithms (GA) effectively resolve conflicts among multiple objectives, enabling optimal trade-offs in multi-parameter optimization and offering a practical framework for multi-objective decision-making [16].

Despite the significant influence of chip morphology on drilling performance, research focused on multi-objective optimization of chip evacuation in gun drilling of oxygen-free copper remains scarce. This paper presents a comprehensive study on the multi-objective optimization of gun drilling process parameters for oxygen-free copper utilizing RSM and GA. By analyzing the effects of feed rate, cutting speed, and cutting fluid pressure on chip morphology indicators—such as chip evacuation coefficient (CCR) and chip volume ratio (R)—this research aims to establish an optimal set of parameters that enhance drilling efficiency and chip removal performance.

## 2. Experimental Methods

**Figure 1:**
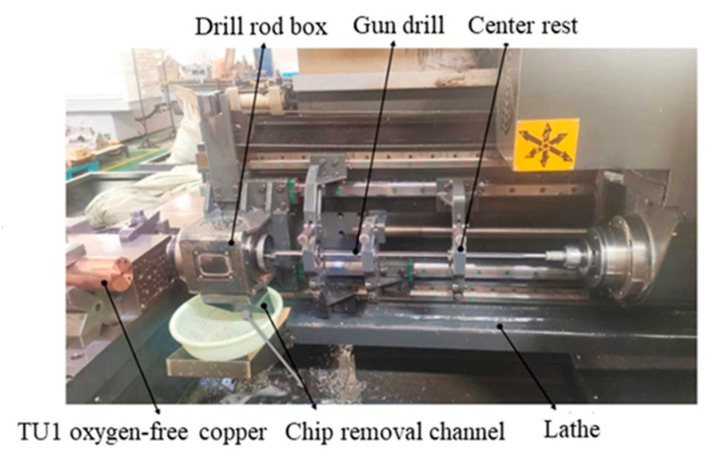
KB1300 three-coordinate deep hole drilling machine.

**Figure 2:**
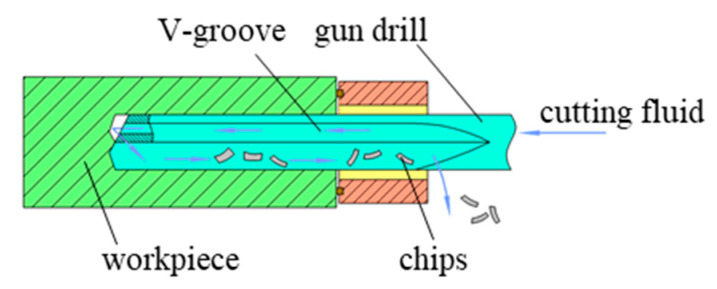
Schematic representation of the gun drilling system.

The gun drilling experiments on oxygen-free copper were conducted using a KB1300 three-coordinate deep hole drilling machine (Shanghai Ningyuan Precision Machinery Corp., Shanghai, China), as shown in Figure 1. The experimental setup comprised a drill rod box, gun drill, center rest, and oil pump, forming a complete gun drilling system. A schematic diagram of the system is presented in Figure 2. Cutting fluid was delivered through the hollow body of the drill directly to the cutting edge, while chip evacuation occurred via the V-shaped flute through hydraulic flushing. ZR-106-emulsified cutting oil (Jiangsu Zirun Chemical Co., Ltd., Nanjing, China) was used as the cutting fluid.

The workpiece material consisted of TU1 oxygen-free copper rods, each measuring 80 mm in diameter and 500 mm in length. The chemical composition and mechanical properties of TU1 copper are detailed in Table 1 and Table 2, respectively. A total of three specimens were used, with nine holes drilled per specimen. Each hole had a diameter of 10 mm and a length-to-diameter ratio of 50.

**Figure 3:**
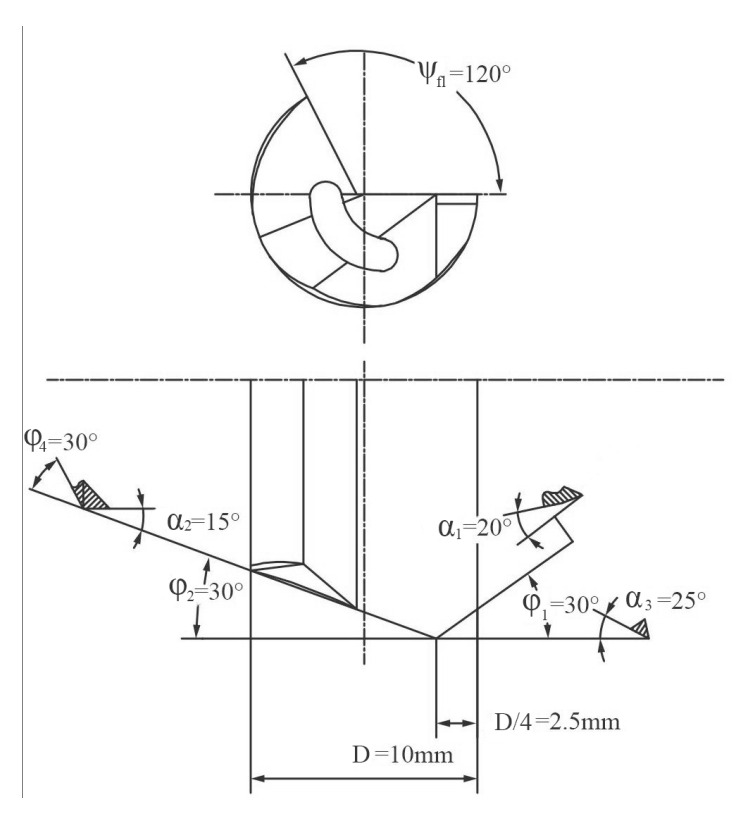
Geometric parameters of the gun drill.

Drilling was performed using a Botek 113 monolithic carbide gun drill with a diameter of 10 mm, manufactured by Botek Präzisionsbohrtechnik GmbH in Riederich, Germany. The geometric parameters of the gun drill are illustrated in Figure 3. The φ_1_ is the outer approach angle, φ_2_ is the inner approach angle, α_1_ is the normal lank angle of the outer cutting edge, α_2_ is the normal flank angle of the inner cutting edge, α_3_ is the normal lank angle of the auxiliary flank surface, φ_2_ is the shoulder dub-off angle, and Ψ_fl_ is the profile angle of the V-groove flute.

To analyze the influence of process parameters on chip morphology during oxygen-free copper gun drilling, the BBD response surface analysis method was utilized for experimental design. The input variables were feed rate, cutting speed, and cutting fluid pressure, while the CCR and R served as response values. A three-factor, three-level response surface experiment was formulated. Based on the literature on deep-hole machining and practical experience with oxygen-free copper processing [18,19,20], the feed rate was varied between 0.012 mm/r and 0.024 mm/r, cutting speed between 47.124 m/min and 62.832 m/min, and cutting fluid pressure between 1.8 MPa and 2.4 MPa. The specific factor levels for the experiment are detailed in Table 3.

## 3. Theoretical Calculation of Chip Morphology Indicators

Chip morphology is a critical factor influencing the efficient evacuation of oxygen-free copper chips during drilling. In this study, chip morphology is assessed using two key indicators: the chip volume ratio (R) and the chip evacuation ratio (CCR). The parameter R quantifies the ability of the drilling process to contain and discharge chips effectively, serving as a measure of chip evacuation performance. CCR, on the other hand, characterizes the extent of chip deformation during cutting, reflecting the material’s plastic flow behavior under shear stress. Together, R and CCR provide a comprehensive evaluation of deformation efficiency and chip removal capability, offering insight into the overall effectiveness of the drilling process.

### 3.1. Calculation of R

The R is a critical metric for assessing chip evacuation efficiency, influencing chip storage space, removal smoothness, and operational safety. It is defined as the ratio of chip volume to the volume of removed metal:

(1) $$R=V_{q}/V_{j}$$

where *V_q_* represents the volume needed for randomly arranged metal chips in mm^3^ and *V_j_* represents the volume of the same amount of metal removal in mm^3^.

Based on prior drilling studies of oxygen-free copper, gun-drilled chips are predominantly spiral. The volume of the spiral chips is calculated as follows:

(2) $$V_{q}=\pi r^{2}l=\pi \frac{1}{K^{2}}wxG$$

where *r* represents the radius of spiral chips in mm; *l* represents the length of the spiral chips in mm; *K* represents the chip curvature radius in mm; *ω* represents the chip width in mm; and *x* is the number of helical turns per chip, which is calculated by Formula (3). *G* is the chip breaking frequency per unit time, which is calculated by Formula (4).

(3) $$x=\frac{Kl_{ch}}{2\pi }$$

where *l_ch_* represents the corresponding uncut chip length in mm.

(4) $$G=\frac{v_{c}}{l_{o}}$$

where *v_c_* represents cutting speed in m/min. *l_o_* represents the total length of chips in mm.

*V_j_* is calculated is calculated by the following formula:

(5) $$V_{q}=1000a_{p}fv_{c}$$

where *a_p_* represents depth of cut in mm and *f* is the feed rate in mm/r.

Therefore, the R is calculated by the following formula:

(6) $$R=\frac{\omega \times \xi_{a}}{2000\times K\times a_{p}\times f}$$

where *ξ_a_* represents the chip compression ratio, which is the ratio of *l_ch_* to *l_o_*.

To account for the influence of cutting conditions on chip formation, a correction factor is introduced into the formulation, yielding the following expression for R:

(7) $$R=A_{v}\frac{\omega \times \xi_{a}}{2000\times K\times a_{p}\times f}$$

where *A_v_* is the correction factor related to workpiece material and cutting parameters. The R is obtained by statistically analyzing chip morphology under varying cutting conditions.

### 3.2. Calculation of CCR

**Figure 4:**
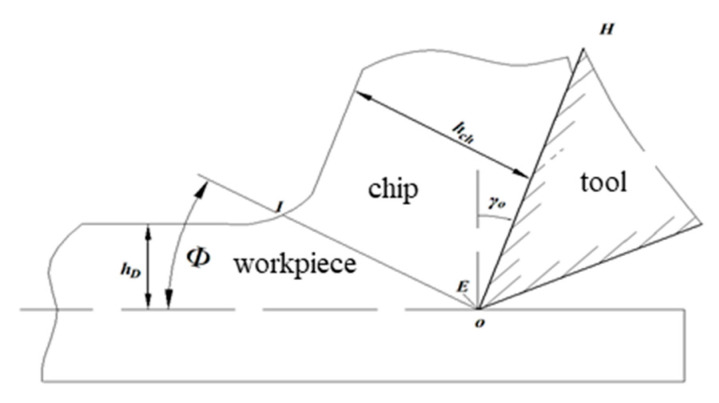
Schematic representation of Chip Deformation Coefficient.

As noted by Savkovic et al. [21], the CCR is determined using Equation (8).

(8) $$\mathrm{CCR}=h_{ch}/h_{D}$$

where *h_ch_* is the chip thickness in mm and *h_D_* is the cutting layer thickness in mm (as shown in Figure 4).

Given the non-uniform chip morphology generated during machining, multiple chip samples are averaged to enhance measurement accuracy:

(9) $$\bar{h_{ch}}=\frac{1}{n}\sum_{i=1}^{n}h_{ch}$$

where $\bar{h_{ch}}$ represents the actual average cutting layer thickness in mm, *n* represents the number of chip samples, and *i* is the variable in the summation process.

The theoretical chip thickness *h_D_* is expressed as follows:

(10) $$h_{D}=f\cos\varphi_{1}$$

where *φ*_1_ is the outer approach angle in degrees. The *φ*_1_ of the gun drill is 30°.

Therefore, CCR can be accurately calculated by statistically analyzing the chip morphology across different cutting parameters:

(11) $$\mathrm{CCR}=\frac{\bar{h_{ch}}}{h_{D}}=\frac{\frac{1}{n}\sum_{i=1}^{n}h_{ch}}{f\cos\varphi_{1}}$$

## 4. Experimental Results and Analysis

**Figure 5:**
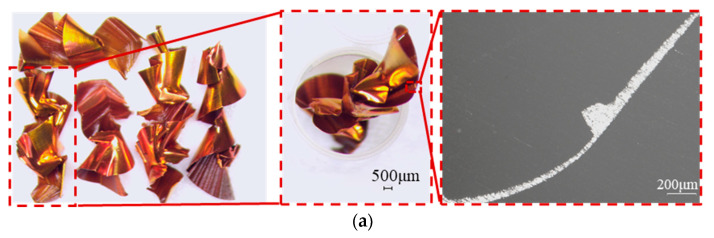

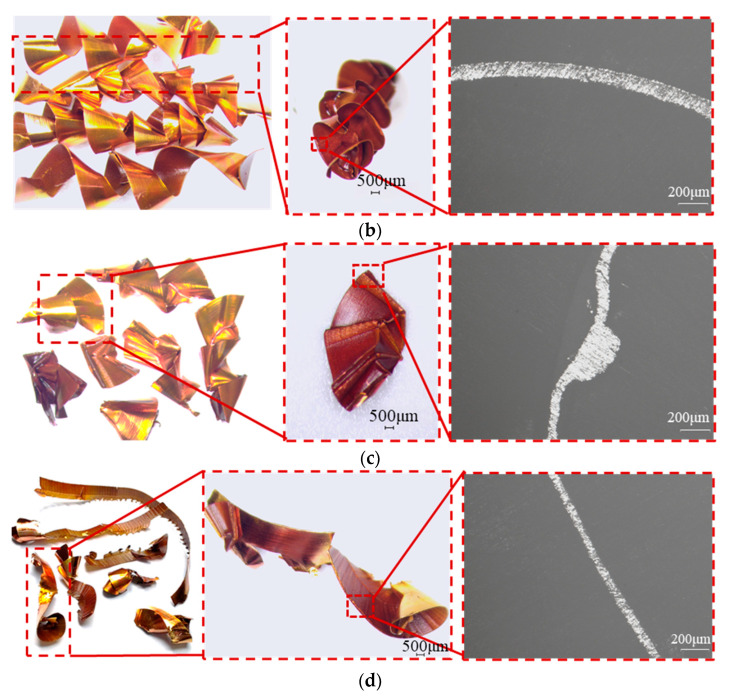
Main chips obtained by RSM: (**a**) extruded spiral chips (**b**) pagoda-like chips; (**c**) C-shaped chips and spiral chips; and (**d**) ribbon chips.

Based on the BBD response surface methodology, 17 sets of experiments were designed, and the resulting chip morphologies were analyzed. The main chip morphology is shown in Figure 5. Chip widths were measured using the Soptop SZM7045 microscope (Sunny Group Co., Ltd., Yuyao, China). Chip thickness and curvature radius were measured using the Lecia MEF 4M microscope (Leica Microsystems, Wetzlar, Germany), and CCR and R were calculated according to Equations (1)–(11), as shown in Table 4.

### 4.1. Analysis of Chip Morphology

The chip morphology under different parameters primarily consists of spiral curls, C-shaped chips, and ribbon chips. When the feed rate is 0.012 mm/r, under varying cutting speeds, the chip morphology predominantly features spiral chips with a pagoda-like shape (NO 2, 12, 13, 14), as shown in Figure 5a. As the cutting speed increases, the length and proportion of long spiral chips also increase. This phenomenon is attributed to the rise in drilling temperature and limited chip evacuation space. Higher cutting speeds elevate the cutting temperature, increasing the plasticity of oxygen-free copper and making chip fragmentation more difficult. These observations align with the findings reported in reference [22] and are attributed to the intrinsic characteristics of oxygen-free copper. Additionally, the confined chip evacuation space and high-speed operation result in chip accumulation and squeezing, leading to elongated chips with significant deformation.

When the feed rate is 0.018 mm/r, the chip morphology across different cutting speeds mainly consists of extruded spiral chips (NO 1, 4, 6, 7, 9, 10, 11, 16, 17), as shown in Figure 5b. As the cutting speed increases, long spiral chips become more prevalent. This behavior is influenced by the material characteristics of TU1 oxygen-free copper. A higher cutting speed generates a greater volume of chips within the same time frame. As the chip volume per unit increases, the chips experience higher forces while passing through the evacuation channel, resulting in increased deformation and longer chip formation.

When the feed rate is 0.024 mm/r, the chip morphology under varying cutting speeds is primarily composed of C-shaped chips and spiral chips (NO 3, 5, 8), as shown in Figure 5c. At higher spindle speeds, ribbon chips are also observed (NO 15), as shown in Figure 5d. This occurs because an increase in both cutting speed and feed rate leads to greater cutting thickness, allowing more metal to flow into the chips within the same time period and subsequently increasing the curvature radius.

### 4.2. Development of Regression Models

Based on the data obtained in Table 4, response surface analysis was conducted to obtain the response surface models for the R and CCR, as shown in Equations (12) and (13).

(12) $$\begin{array}{r} y_{R}=19884.375f^{2}+0.060684v^{2}-43.07819P^{2}-10,141.26458f-26.6954v\\ -215.78492P+213.8109fv-2025.13889fP+8.24643vP+1044.11956 \end{array}$$

(13) $$\begin{array}{r} y_{\mathrm{CCR}}=10146.94f^{2}+0.000769v^{2}+0.90906P^{2}-664.38167f-1.65948v\\ -41.2941P+4.40911fv-206.79167fP+0.76691vP+101.65697 \end{array}$$

where y_R_ represents the response surface models of chip volume ratio; y_CCR_ represents the response surface models of the chip evacuation coefficient; and feed rate, cutting speed, and cutting fluid pressure are independent variables, denoted as *f*, *v*, and *P*, respectively.

The analysis of variance (ANOVA) for the response surface models of R and CCR was conducted based on the established regression models, as shown in Table 5. The regression equation coefficients (R^2^), F-value, and *p*-value were analyzed to evaluate the suitability and significance of the response model. According to ANOVA theory, if the F-value is greater than 1 and the *p*-value is less than 0.05, the developed model is considered valid [23]. The R^2^ values for the CCR and R are 0.9411 and 0.9626, respectively, with F-values of 12.42 and 20.01 and *p*-values both less than 0.05, indicating that the prediction models are significant. Further analysis of *p*-values in the response surface model for CCR reveals the relationship P_f_ (0.0001) < P_v_ (0.0254) < P_P_ (0.3968), indicating that the influence of the three single factors on the CCR, ranked in descending order, is feed rate, cutting speed, and cutting fluid pressure. Similarly, examining the *p*-values in the response surface model for R shows P_f_ (0.0002) < P_v_ (0.0011) < P_P_ (0.0088), leading to the conclusion that the influence of the three single factors on the R, in descending order, is feed rate, cutting speed, and cutting fluid pressure.

### 4.3. Analysis of Single and Interactive Effects of Process Parameters on CCR and R

**Figure 6:**
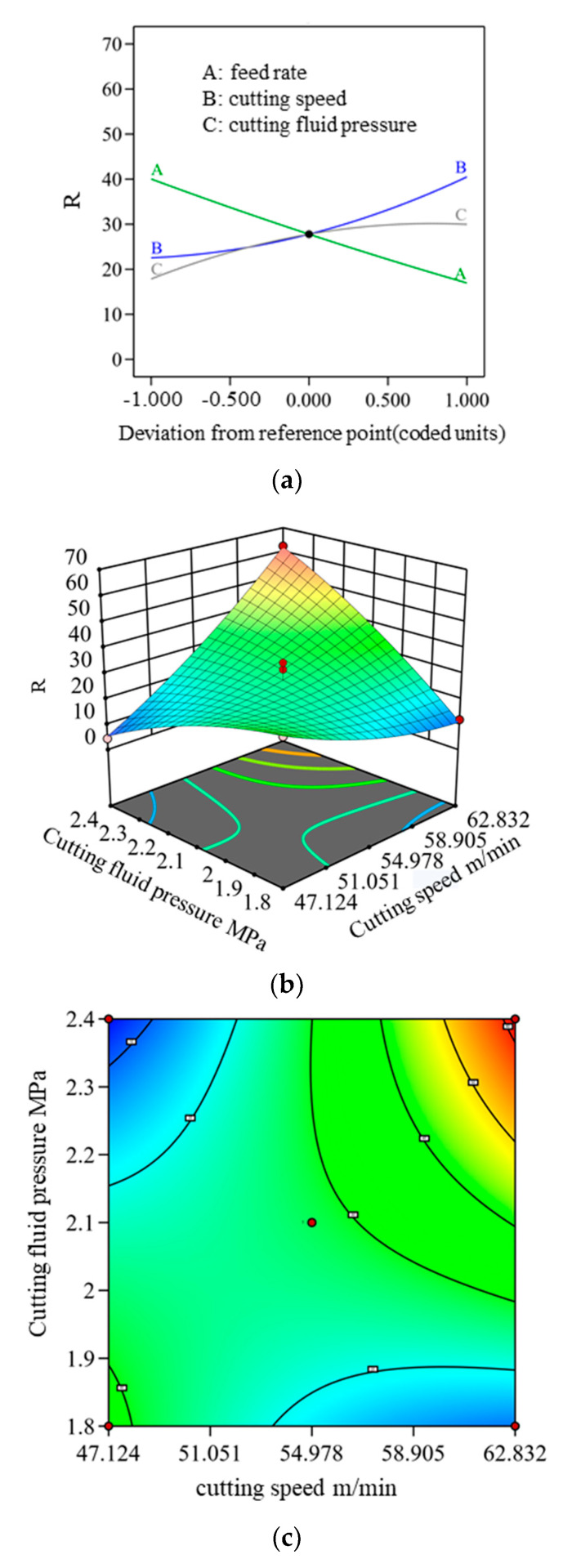
Influence of individual and combined gun drilling parameters on R: (**a**) influence of single factors on R; (**b**) 3D surface plot of the interaction between cutting fluid pressure and cutting speed; (**c**) contour plot of the interaction between cutting fluid pressure and cutting speed.

**Figure 7:**
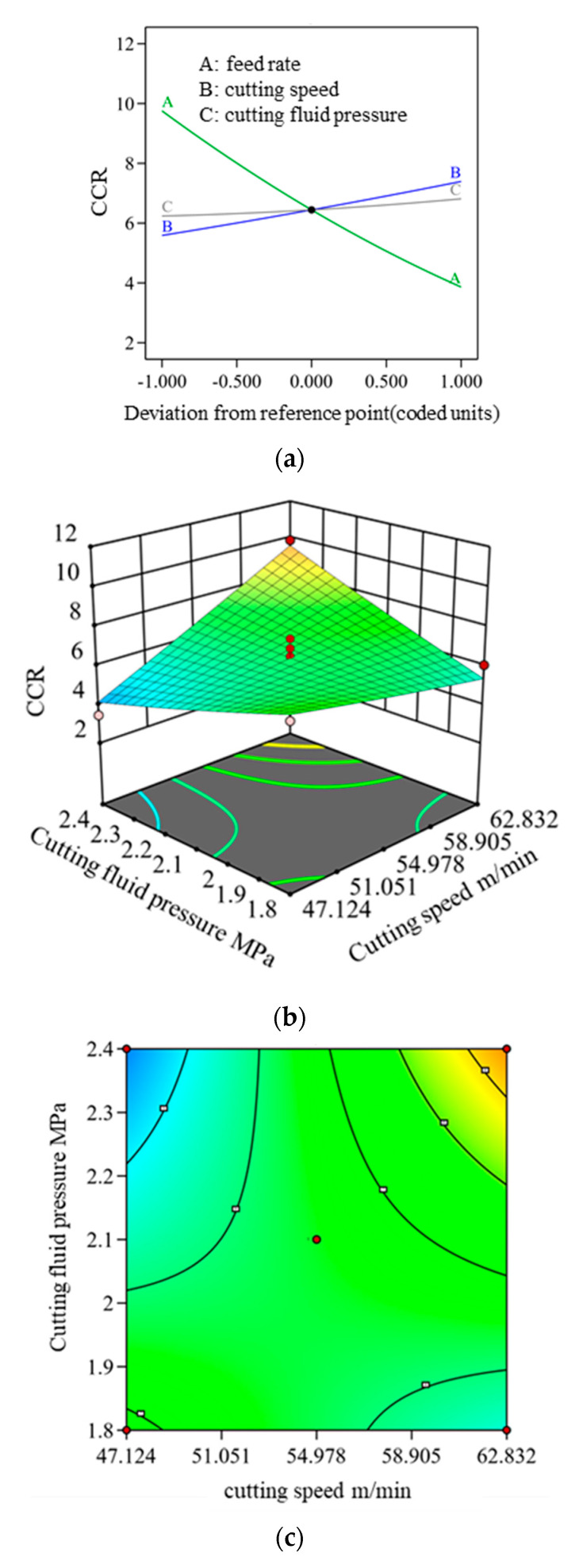
Influence of individual and interactive gun drilling parameters on the CCR: (**a**) Influence of single factors on CCR; (**b**) 3D surface plot of interaction between cutting fluid pressure and cutting speed; (**c**) contour plot of interaction between cutting fluid pressure and cutting speed.

To better visualize the single and interactive effects of feed rate, cutting speed, and cutting fluid pressure on CCR and R, single-factor influence diagrams, 3D Surface plots, and contour plots were generated, as shown in Figure 6 and Figure 7.

Figure 6a illustrates the impact of individual cutting parameters on R. Both cutting speed and cutting fluid pressure exhibit a positive correlation with R, with cutting speed exerting a more pronounced effect. Conversely, feed rate demonstrates a negative correlation with R.

In the interaction variance analysis, P_AB_ (0.0039) and P_BC_ (<0.0001) are both less than 0.05, indicating a significant interaction effect between cutting speed and cutting fluid pressure on R when multiple factors are combined. The interaction between cutting speed and feed rate also contributes notably. Figure 6b,c illustrate the interactive influence of cutting speed and cutting fluid pressure. The data indicate that both parameters increase along the diagonal axis, with contour lines becoming progressively denser, signifying that R rises as cutting speed and cutting fluid pressure increase. This trend is primarily driven by the temperature sensitivity of oxygen-free copper. Higher cutting speeds lead to elevated cutting temperatures, causing the material to expand and deform, making chip fragmentation more challenging [22]. Additionally, a greater volume of metal is removed within the same time frame, resulting in chip squeezing and accumulation. As cutting fluid pressure increases, the force exerted on the chips also rises. However, due to the high plasticity of oxygen-free copper, chip deformation intensifies, leading to an increase in R. To optimize chip morphology and control deformation, selecting an appropriate cutting speed and cutting fluid pressure range under multi-factor interactions is essential.

In machining operations, CCR is a key parameter for assessing chip morphology quality. Figure 7a illustrates the impact of individual factors on the CCR, showing that cutting speed and cutting fluid pressure exhibit a positive correlation with CCR, whereas feed rate has a negative correlation. The interaction variance analysis indicates that P_BC_ (0.0052) is less than 0.05, confirming that the interaction between cutting speed and cutting fluid pressure has a significant influence on CCR when multiple factors are considered. Figure 7b,c present the interactive effects of cutting speed and cutting fluid pressure. The data demonstrate a diagonal increase, with contour lines becoming progressively denser, signifying that CCR rises with increasing cutting speed and cutting fluid pressure. This trend aligns with the factors affecting R, primarily driven by the thermal sensitivity and plasticity of oxygen-free copper, which leads to increased chip deformation and evacuation challenges.

## 5. GA Optimization and Validation

### 5.1. GA Optimization

Based on the response surface models for R and CCR presented in Equations (12) and (13), a multi-objective optimization was performed using a GA, aiming to minimize both R and CCR. The optimization focused on three key process parameters—feed rate, cutting speed, and cutting fluid pressure—each constrained within specific ranges: feed rate from 0.012 mm/r to 0.024 mm/r, cutting speed from 47.124 m/min to 62.832 m/min, and cutting fluid pressure from 1.8 MPa to 2.4 MPa.

**Figure 8:**
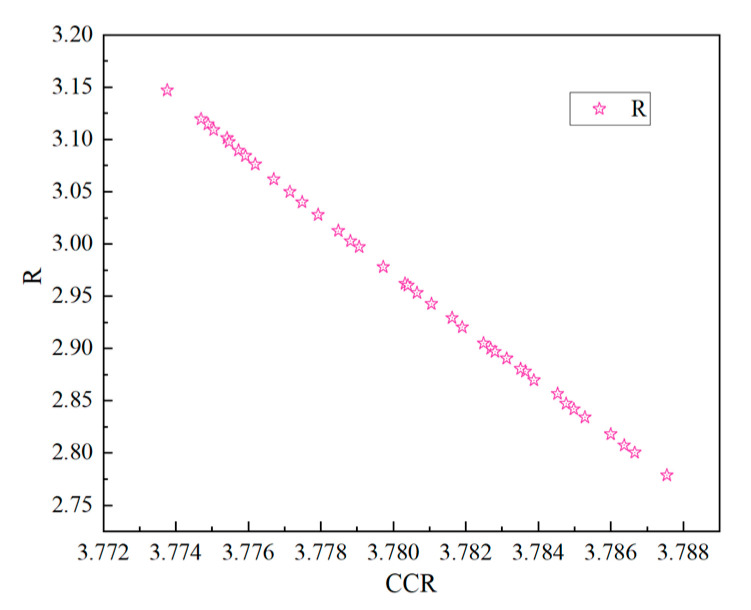
Pareto Front between R and CCR.

The GA was configured with 200 iterations, a crossover factor of 0.8, a mutation probability of 0.2, and both crossover and mutation distribution indices set to 20. Under these settings, the algorithm iteratively refined the parameter values, generating a Pareto front (Figure 8) that illustrates the trade-off between R and CCR. This front represents the set of optimal solutions that balance the two objectives, highlighting the feasible combinations that offer the best compromise. After more than 200 iterations, the results stabilized, and multiple optimized parameter sets were obtained. Given that R plays a critical role in ensuring smooth chip evacuation, solutions with lower R values were prioritized. The final optimized configuration yielded a feed rate of 0.0189 mm/r, a cutting speed of 47.4381 m/min, and a cutting fluid pressure of 2.3979 MPa, resulting in a CCR of 3.7875 and an R of 2.7788.

### 5.2. Experimental Validation

To validate the parameter set optimized via the RSM-GA approach, gun drilling experiments were performed under actual machining conditions. While the RSM-GA yields an idealized solution, the exact replication of the optimized parameters is often constrained by physical and practical limitations. Therefore, a feasible parameter set was selected to closely approximate the optimized values: a feed rate of 0.019 mm/r, a cutting speed of 47.1 m/min, and a cutting fluid pressure of 2.4 MPa.

**Figure 9:**
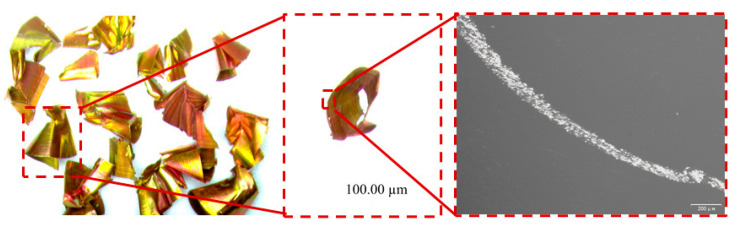
Chip morphology under GA optimized parameters.

Under these conditions, the resulting CCR was 3.2951, and the R value was 3.3345. The chips formed were predominantly C-shaped, promoting efficient evacuation, as shown in Figure 9.

## 6. Conclusions

This study employed a combination of response surface methodology and genetic algorithm to optimize the drilling process parameters for porous TU1 oxygen-free copper, focusing on their impact on R and CCR. The key findings are as follows.

(1) Response surface variance analysis revealed that, in single-factor influence assessments, feed rate had the most significant effect on R and CCR, followed by cutting speed, with cutting fluid pressure exerting the least influence. In multi-factor interactions, the combination of cutting speed and cutting fluid pressure had the most substantial impact on CCR and R.
(2) Considering actual machining conditions and the genetic algorithm optimization results, the optimal drilling process parameters were determined to be a feed rate of 0.019 mm/r, a spindle speed of 47.1 m/min, and a cutting fluid pressure of 2.4 MPa. Under these conditions, the CCR was 3.2951, and R was 3.3345, with chips primarily exhibiting a C-shaped morphology and smooth chip evacuation.

Through comprehensive experimentation and analysis, this study offers valuable insights into the interplay between drilling parameters and chip morphology. It establishes a robust optimization framework applicable to various industrial scenarios, particularly for enhancing drilling efficiency and chip evacuation in materials with high plasticity.

Nonetheless, several limitations remain. The accuracy of the RSM model is highly sensitive to the number, distribution, and range of sample points used in the initial experimental design. Inadequate or poorly distributed sampling can result in suboptimal global modeling, and predictions from the RSM model tend to be unreliable outside the sampled region. Furthermore, if the current RSM model exhibits significant error, the genetic algorithm may be misdirected—prematurely converging to a pseudo-optimal solution based on the flawed model rather than identifying the true global optimum.

## Figures and Tables

**Table 1:** Chemical composition (wt.%) [17].

Cu+Ag	P	Bi	Sb	As	Fe	Ni	Pb	Sn	S	Zn	O
99.97	0.002	0.001	0.002	0.002	0.004	0.002	0.003	0.002	0.004	0.003	0.002

**Table 2:** Mechanical properties of TU1 oxygen-free copper.

Yield Strength (MPa)	Tensile Strength (MPa)	Thermal Conductivity (W/mK)	Coefficient of Linear Thermal Expansion (°C)
49–78	215–254	391	18.6 × 10^−6^

**Table 3:** Factor ranges and levels.

Level	Factors		
	Feed Rate (mm/r)	Cutting Speed (m/min)	Cutting Fluid Pressure (MPa)
−1	0.012	47.124	1.8
0	0.018	54.978	2.1
1	0.024	62.832	2.4

**Table 4:** Response surface design and results for TU1 oxygen-free copper gun drilling.

No	Feed Rate (mm/r)	Cutting Speed (m/min)	Cutting Fluid Pressure (MPa)	CCR	R
1	0.018	47.124	1.8	6.8974	31.7415
2	0.012	47.124	2.1	9.6923	43.1025
3	0.024	47.124	2.1	3.2019	4.6235
4	0.018	47.124	2.4	3.4583	4.3855
5	0.024	54.978	1.8	3.9211	7.9765
6	0.018	54.978	2.1	6.932	34.559
7	0.018	54.978	2.1	6.5231	31.8955
8	0.024	54.978	2.4	4.1534	13.4295
9	0.018	54.978	2.1	7.423	26.411
10	0.018	54.978	2.1	6.3675	22.8165
11	0.018	54.978	2.1	4.975	23.219
12	0.012	54.978	1.8	8.8846	28.518
13	0.012	54.978	2.4	10.606	48.552
14	0.012	62.832	2.1	10.096	39.704
15	0.024	62.832	2.1	4.4369	41.5275
16	0.018	62.832	1.8	6.0744	12.047
17	0.018	62.832	2.4	9.8633	62.412

**Table 5:** ANOVA for R and CCR.

Source	CCR			R		
	F-Value	*p*-Value		F-Value	*p*-Value	
Model	12.42	0.0016	Significant	20.01	0.0003	Significant
A-f	85.26	<0.0001	Significant	46.89	0.0002	Significant
B-v	8.01	0.0254	Significant	28.39	0.0011	Significant
C-P	0.8145	0.3968		12.94	0.0088	Significant
AB	0.2121	0.6591		17.87	0.0039	Significant
AC	0.6807	0.4365		2.34	0.1700	
BC	16.04	0.0052	Significant	66.46	<0.0001	Significant
A^2^	0.6901	0.4335		0.0950	0.7669	
B^2^	0.0116	0.9172		2.60	0.1511	
C^2^	0.0346	0.8577		2.79	0.1391	
Residual						
Lack of Fit	0.9237	0.5062		0.5062	0.6529	

## Data Availability

The original contributions presented in this study are included in the article. Further inquiries can be directed to the corresponding authors.
